# 619 Pain Management in Burn and Wound Patients: Effects of Acupuncture in Pain Score and Medication

**DOI:** 10.1093/jbcr/irac012.247

**Published:** 2022-03-23

**Authors:** Erika Tay Lasso, Anjali Herekar, Clara Riggle, Sheng Li, Hui Hwang, Mariko Horie, Kenneth S Phelps, Kimberly Burton, Leonardo Alaniz, Gabrielle Hovis, Kim Hecht, Molly E Nunez, Victor C Joe, Theresa L Chin

**Affiliations:** University of California Irvine Medical Center, Orange, California; University of California Irvine School of Medicine, Orange, California; University of California school of medicine, Orange, California; University of California Irvine Medical Center, Orange, California; University of California Irvine medical center, orange, California; University of California Irvine Medical Center, Orange, California; University of California Irvine Medical Center, Huntington Beach, California; University of California Irvine, Orange, California; University of California Irvine Medical Center, Orange, California; University of California Irvine School of Medicine, Orange, California; University of California Irvine Medical Center, orange, California; UCI Susan Samueli Integrative Health Institute, Orange, California; UCI Health Regional Burn Center, Orange, California; University of California Irvine, Orange, California

## Abstract

**Introduction:**

Pain management in burn patients is a complex ongoing problem. Opioid and non-opioid medications have historically been used as the primary pain management modality in burn care. Considering the ongoing opioid epidemic, a multidisciplinary holistic approach is needed for pain management. Acupuncture has been recognized to help in different settings such as chronic back pain, neurological problems, depression, and anxiety.

We hypothesize that burn and wound patients receiving acupuncture will have a decrease in opioids and non-opioid medications on acupuncture day (ACd) compared to non-acupuncture day (n-ACd) and a reduction of pain scores before and after acupuncture.

**Methods:**

A prospective observational study in all patients admitted to the burn service was made. We exclude patients that were unable to provide consent or age 6 years and younger.

A numeric pain score (1-10) was collected from the patients before and after each acupuncture session; if patients were sleeping after acupuncture pain score was collected when they woke up. A prospective collection of daily opioids and non-opioids medications was made. Opioids were converted to daily morphine equivalents (MME).

**Results:**

From February to August 2021, 53 patients on the burn service were treated with acupuncture for a total of 185 sessions. The median age was 49 years (IQR:32.5-63.0). Median TBSA for burn patients was 3.3% (IQR:1.5-8.1). The number of sessions per patient ranged from 1-12 with a median of 3 (IQR1.7-4). Before acupuncture, the median pain score was 5 (IQR:2.6-6.4), and after treatment was it was 1.5 (IQR:0.2-2.8).

Of 185 sessions, 49 (26%) of those sessions, patients fell asleep afterward. Median morphine use on ACd was 26.6 MME (15.8-62.1) vs. n-ACd 27.35 MME (15.5-65.3). For non-opioid medication, the median of acetaminophen on ACd 1007.5mg (313.39 -1950) vs. n-ACd 1381.25mg (642.5-2535).

After adjusting for TBSA and chronic opioid use, patients were given a daily mean of 391 MME CI: (1201.12-419.12 p=0.334) less in ACd vs. n-ACd. In addition, acetaminophen had a daily mean of 395.12mg CI: (130.05, 660.19 p=0.005) less in ACd vs. n-ACd.

Overall, acupuncture was associated with a significant lower pain score with a mean of 2.799 points less.

**Conclusions:**

Acupuncture seems to abrogate pain in burn and wound patients, as demonstrated by doses of pain medication. While there was no significant difference in opioid medications, there was a significant difference in acetaminophen, indicating that additional education of a tiered approach to pain medications is essential to reduce unnecessary opioid use.

